# Neuropsychological performance in women at risk of postpartum depression and postpartum psychosis

**DOI:** 10.1007/s00737-024-01510-9

**Published:** 2024-08-31

**Authors:** Giulia Cattarinussi, Giulia Segre, Alessandra Biaggi, Katie Hazelgrove, Fabio Sambataro, Manuela Russo, Andrew Lawrence, Montserrat Fusté, Mitul A. Mehta, Gertrude Seneviratne, Michael C. Craig, Maddalena Miele, Susan Pawlby, Susan Conroy, Carmine M. Pariante, Paola Dazzan

**Affiliations:** 1 Department of Psychological Medicine, Institute of Psychiatry, Psychology and Neuroscience, King’s College London, London, UK; 2 Department of Neuroscience (DNS), Padua Neuroscience Center, University of Padova, Padua, Italy; 3 Department of Pharmacological and Biomolecular Sciences, University of Milan, Milan, Italy; 4 Centre for Implementation Science, Health Service and Population Research Department, Institute of Psychiatry, Psychology and Neuroscience, King’s College London, London, UK; 5 National Institute for Health Research (NIHR) Mental Health Biomedical Research Centre at South London and Maudsley NHS Foundation Trust and King’s College London, London, UK; 6 Centre for Neuroimaging Sciences, Institute of Psychiatry, Psychology and Neuroscience, King’s College London, London, UK; 7 South London and Maudsley NHS Foundation Trust Channi Kumar Mother and Baby Unit, Bethlem Royal Hospital, London, UK; 8 National Female Hormone Clinic, Maudsley Hospital, SLaM NHS Foundation Trust, and Institute of Psychiatry, Psychology and Neuroscience, King’s College London, London, UK; 9 Perinatal Mental Health Service, St Mary’s Hospital, Imperial College London and Central North West London NHS Foundation Trust, London, UK

**Keywords:** Cognitive function, Antenatal depression, Postpartum depression, Postpartum psychosis, Childbirth

## Abstract

**Purpose:**

While neuropsychological deficits are commonly observed in affective and psychotic disorders, this remains unexplored in these disorders when they occur during pregnancy and the postpartum period.

**Methods:**

A neuropsychological test battery was administered to women defined at risk of postpartum depression (PD, *N* = 53) because having either a current or past diagnosis of major depressive disorder, women at risk of postpartum psychosis (PP, *N* = 43) because of a diagnosis of bipolar disorder or schizoaffective disorder and/or a previous episode of PP and women not at risk (NR, *N* = 48) in the third trimester of pregnancy. Generalized and specific cognitive abilities were compared between groups.

**Results:**

Women at risk of PP presented worse executive functions and processing speed compared to NR and worse performance compared to women at risk of PD across all cognitive domains. In addition, women at risk of PP who developed a psychiatric relapse in the first four weeks post-partum showed worse verbal learning and memory, visual memory, executive functions and processing speed in pregnancy compared to NR, whereas women at risk of PP who remained well presented neuropsychological performance that was intermediate between that of the women NR and those at risk of PP who developed symptoms. There were no differences in performance between women at risk of PD and the NR women, even if 31 women at risk of PD presented depressive symptoms at the time of cognitive assessment.

**Conclusions:**

Our findings in women at risk of PP align with neuropsychological findings in individuals with, or at risk of psychosis unrelated to pregnancy. In addition, initial evidence that women at risk of PP who develop a psychiatric relapse in the postpartum show a particularly poor neuropsychological performance in pregnancy suggests that this could be considered part of a phenotype for the disease and help guiding future preventive strategies in this clinical population. In women at risk of PD, the presence of depressive symptoms did not influence cognitive performance.

**Supplementary Information:**

The online version contains supplementary material available at 10.1007/s00737-024-01510-9.

## Introduction

The perinatal period is a time of high risk for the onset of psychiatric disorders (Jones 2012). According to the World Health Organization, about 10–15% of women in high income countries are affected by perinatal mental health problems, and the percentage rises to approximately 30% in low- and middle-income countries (World Health Organization, 2008). Perinatal psychiatric disorders are associated with severe morbidity for the mother and the infant, including increased risk of low birthweight and prematurity, less optimal mother-infant relationship and developmental disturbances in the child (Netsi et al. 2018; Stein et al. 2014). Among perinatal mental health problems, depression and anxiety disorders are the most common and they generally present with the same symptoms as in non-childbearing disorders (Howard et al. 2014). For depression, the prevalence of major depressive disorder (MDD) ranges between 3.1% and 4.9% in pregnancy and remains stable in the first 3 months postpartum (Gavin et al. 2005). Women with a prior psychiatric history are particularly at risk of developing a depressive disorder in pregnancy and in the postpartum period (Howard et al. 2013; Lancaster et al. 2010). On the other hand, women with a history of bipolar disorder (BD), schizoaffective disorder (SZA), or a previous episode of PP, are at increased risk of developing postpartum psychosis (PP), a severe mental disorder characterized by irritability, depressed mood, restlessness, confusion, hallucinations, delusions and disorganised behaviour that typically develop within the first four weeks after delivery (Jones et al. 2014; Jones and Craddock 2001; Munk-Olsen et al. 2006). Although the absolute prevalence is low, with 1–2 cases/1000 births in the general population, the risk of developing PP has been reported to be as high as 30–50% in women with a history of affective psychosis (Robertson et al. 2005; Wesseloo et al. 2016).

Cognitive impairments, including deficits in memory, attention and executive functions, have been commonly described in individuals with depression (Kriesche et al. 2023) and psychosis (Ceylan et al. 2020; McCleery and Nuechterlein 2019), as well as in individuals at clinical or genetic risk for these disorders (Cattarinussi et al. 2023; Fusar-Poli et al. 2012; Mackenzie et al. 2019). Crucially, these neuropsychological profiles have also been reported in women with the same mental health disorders across the lifespan (Colenda et al. 2010; Wang et al. 2020; Yaffe et al. 1999). Interestingly, evidence that has emerged over the past few decades has shown that sex hormones can play an important role in cognition in women. In particular, estradiol presents a positive modulatory effect on neuropsychological function due to its neurotrophic effect (Frick 2015; Luine 2014). In addition, other hormones seem to particularly influence cognitive performance in women, including cortisol (Smeets et al. 2009), oxytocin (Kunitake et al. 2022) and thyroid hormones (Grigorova and Sherwin 2012).

While neuropsychological performance in women with psychiatric disorders has been extensively studied, the literature on neuropsychological functioning in women at risk of mental health problems in pregnancy and in the early postpartum period is still limited. A study that examined working memory in pregnant women highlighted that women with depression in pregnancy presented working memory deficits, while working memory was not affected in pregnant women without depressive symptoms (Hampson et al. 2015). Interestingly, in these women higher estradiol levels appeared to be associated with better working memory performance (Hampson et al. 2015). Another study found differences in self-reported memory difficulties in pregnant and postpartum women compared to healthy, never-pregnant female controls, although there were no differences in neuropsychological tests at any time point. Mood and quality of life slightly moderated specific measures of attention and verbal fluency (Logan et al. 2014). To the best of our knowledge, cognitive performance in women at risk of PP during pregnancy and the perinatal period has not been examined so far.

Considering the paucity of evidence, we conducted the first investigation of neuropsychological performance in women at risk of PD (postpartum depression) and women at risk of PP (postpartum psychosis), to better understand the relationship between risk of perinatal psychopathology and neuropsychological performance. Furthermore, we tested whether worse neuropsychological performance during pregnancy predicts the development of psychiatric symptoms in the post-partum. In line with the literature on neuropsychological deficits unrelated to pregnancy, we hypothesized that, in pregnancy, women at risk of PP would present worse neuropsychological performance compared to women not at risk (NR), while women at risk of PD would show a neuropsychological performance intermediate between that of women at risk of PP and that of women NR. In addition, we hypothesized that a poorer neuropsychological performance in pregnancy would be associated with the development of psychiatric symptoms in the post-partum.

## Materials and methods

### Design

This sample is part of the Psychiatry Research and Motherhood (PRAM) study, a prospective longitudinal study that recruited and followed up women with a DSM-IV diagnosis of MDD during pregnancy or at risk of perinatal depression, women at risk of developing PP (see below) and a control group of pregnant women with no current or previous psychiatric diagnosis, from the second/third trimester of pregnancy up to one year postnatally. At baseline (25–30 weeks gestation), we assessed maternal socio-demographic factors, obstetric and physical risk factors, as well as clinical status. At around 30 weeks gestation, a neuropsychological test battery was administered to participants to assess cognitive function. Maternal clinical status was re-assessed in the first weeks post-partum to establish whether women at risk of PD and PP developed a psychiatric relapse after the baseline evaluation. All participants gave written consent and the studies were approved by the King’s College Hospital Research Ethics Committee (approval number REC 07/Q0703/48) and the Camberwell St Giles. Ethics Committee (REC: 10/H0807/14).

### Sample

The sample included pregnant women at risk of postpartum depression (PD) because having either a current diagnosis of MDD or having a previous history of MDD while currently well; and a group of women currently well but considered at risk of PP because of a diagnosis of bipolar disorder or schizoaffective disorder and/or a previous episode of PP (at risk of postpartum psychosis - PP). We also recruited a group of pregnant women not at risk (NR), with no current or previous history of mental health disorders and no family history of postpartum psychosis. Women were: (a) in the late second or third trimester of a singleton pregnancy; (b) aged at least 18 years; (c) native speakers of English or with a good command of English as a non-native language (see Supplementary Material for exclusion criteria). A total of 144 women were included in the current analyses (At risk of PP *n* = 43, At risk of PD *n* = 53, NR *n* = 48). In the first four weeks post-partum, 18 women at risk of PP developed a psychiatric relapse (At risk of PP-unwell) and 25 remained well (At risk of PP-well).

### Clinical assessment

At baseline (25–30 weeks gestation), all participants were assessed for current and past psychiatric disorders using the Structured Clinical Interview for DSM-IV (SCID I – CV) (First 1996). We additionally used the Positive and Negative Syndrome Scale (PANSS) (Kay et al. 1987), the Young Mania Rating Scale (YMRS) (Young et al. 1978) and the Hamilton Depression Scale (HAM-D) (Hamilton 1960) to assess psychotic and mood symptoms. Global functioning was tested with the Clinical Global Impression Scale (CGI) (Busner and Targum 2007) and the General Assessment of Functioning (GAF) (Aas 2010). Women at risk of PD and PP were also followed up to establish the presence of any psychiatric relapse (see Supplementary Material for definition of postpartum psychosis).

### Neuropsychological assessment

At 30 weeks of pregnancy (30.2 ± 3.7, range: 21.7–39.1 weeks), we evaluated seven neurocognitive domains, using the Wechsler Adult Intelligence Scale – Revised (WAIS-R) (Wechsler 1981), Wechsler Test of Adult Reading (WTAR) (Wechsler 2001) and Wechsler Memory Scale-III (WMS-III) (Wechsler 1997) (see Supplementary Material). With the exception of scores that are already standardized based on population norms (FSIQ, WTAR), z-scores for the neurocognitive measurements were created using the normative standards from the control sample. Only the Trail Making Test B presented significant skewness, so we decided to replace outliers (mean ± 3 standard deviations) with the value equivalent to mean ± 3 standard deviations (Zanelli et al. 2013).

### Statistical analysis

First, we compared demographic, clinical and neuropsychological data in women at risk of PD, women at risk of PP and NR. Then, since only women at risk of PP showed a difference in neuropsychological performance compared to NR, we further explored differences between those women at risk of PP who developed a psychiatric relapse in the first four weeks postpartum (At risk of PP-unwell), the women at risk of PP who remained well (At risk of PP-well) and the NR. Between-group differences were tested using t-test or one-way ANOVA, Chi-square, Mann-Whitney or Kruskal-Wallis tests, as appropriate. Normality was tested with the Shapiro Wilk test. Significance was set at a two-tailed p-value < 0.05. Univariate covariance analysis (ANCOVA) and generalized linear model were used to compare the neuropsychological performance between the three groups, with level of education as covariate for parametric and non-parametric data, respectively. Post-hoc tests were then performed when significant differences were found, using Tukey correction for ANOVA and Bonferroni-Holm correction for ANCOVA and generalized linear model. We evaluated correlations using Pearson and Spearman coefficients, for parametric and non-parametric data, respectively. Statistical analyses were performed with the SPSS 29.0.1.0 and Jamovi 2.3.21.0.

## Results

### Demographic and clinical characteristics

There were no significant differences in age, ethnicity and parity among women at risk of PD, women at risk of PP and NR (Table 1). The groups differed significantly in level of education (χ2 = 7.88, df = 2, *p* = 0.019) and employment (χ2 = 10.7, df = 3, *p* = 0.03), with a higher proportion of women at risk of PD presenting a lower level of education and employment compared with women at risk of PP and NR. Results of the Kruskal-Wallis test showed that women at risk of PD and women at risk of PP presented worse PANSS, HAM-D, CGI and GAF scores compared to NR. In addition, women at risk of PP had higher YRMS scores compared to women NR. There were no differences in clinical scales scores between women at risk of PP and women at risk of PD (all p’s > 0.05) (Table 1).

**Table 1:** Demographic and clinical variables of AR-PD, AR-PP and NR

	AR-PD *n* = 53	AR-PP *n* = 43	NR *n* = 48	Statistics	*p*-value
Age (years), m (SD)	32.3 (6.7)	32.3 (5.8)	32.9 (4.6)	K = 0.378	0.828
Ethnicity (Caucasian), n (%)	41 (77.4%)	27 (62.8%)	38 (79.2%)	χ²(2) = 12.4	0.054
Parity (primiparity), n (%)	32 (60.4%)	27 (62.8%)	25 (52.1%)	χ²(3) = 4.38	0.357
Educational level Degree/diploma Vocational/college or lower	* 31 22	32 11	40 8	χ²(2) = 7.88	**0.019**
Employment level Professional/managerial Non-professional/non-managerial Never employed	* 26 22 5	21 19 3	37 9 2	χ²(3) = 10.7	**0.030**
Diagnosis	MDD	34 BD, 5 SZA, 4 PP	N/A	N/A	N/A
Medications (yes), n (%) ^a^	12 (22.6%)	21 (48.8%)	N/A	χ²=7.22	**0.007**
PANSS Total score, m (SD) ^b^	34.0 (5.0)*	34.5 (6.7)*	30.4 (1.1)	K = 30.317	**< 0.001**
YMRS Total score, m (SD) ^b^	1.3 (1.8)	1.9 (2.3)*	0.65 (1.3)	K = 9.218	**0.01**
HAM-D Total score, m (SD) ^b^	7.8 (7.2)*	6.5 (5.1)*	2.8 (2.5)	K = 18.335	**< 0.001**
CGI Total score, m (SD) ^b^	2.9 (1.0)*	2.7 (0.8)*	2.0 (0.0)	K = 37.047	**< 0.001**
GAF Total score, m (SD) ^b^	79.5 (10.6)*	75.1 (10.6)*	91.5 (4.2)	K = 65.853	**< 0.001**

AR-PD: women at risk of perinatal depression; AR-PP: women at risk of post-partum psychosis; BD: bipolar disorder; CGI: clinical global impression; GAF: global assessment of functioning; HAM-D: Hamilton Depression Rating Scale; MDD: major depressive disorder; m: mean; N/A: not applicable; NR: women not at risk; PANSS: Positive and Negative Syndrome Scale; PP: previous episode of postpartum psychosis; SD: standard deviation; SZA: schizoaffective disorder; YMRS: Young Mania Rating Scale

^a^ third trimester

^b^ baseline (25–30 weeks gestation)

*p*-values refer to the comparison between AR-PD, AR-PP and NR

* Significant results of post-hoc analyses between AR-PD and NR and between AR-PP and NR

Higher PANSS, YMRS, HAM-D and CGI scores indicate more severe symptoms, while higher GAF scores indicate better functioning

In the overall sample, as well as in each group, scores of the PANSS, YRMS, HAM-D and CGI scales were not correlated with neuropsychological performance scores, while GAF scores were positively correlated with verbal learning and memory, executive functions and processing speed (all p’s < 0.001, Table S1). Among women at risk of PP, average antipsychotic dose in the third trimester of pregnancy did not differ between At risk of PP-unwell and At risk of PP-well women. There was no difference between the At risk of PP-unwell and At risk of PP-well women in level of education.

Women at risk of PP who became unwell in the post-partum already had worse scores across all clinical scales compared to NR at baseline. Similarly, women at risk of PP who remained well in the post-partum also had worse baseline symptoms than NR across these scales, with the exception of the YMRS. In addition, women at risk of PP who became unwell in the post-partum already had higher PANSS and HAM-D scores at baseline compared to women at risk of PP who remained well in the post-partum (Table 2). After correction for multiple comparisons, we found no correlation between PANSS, YRMS, HAM-D and CGI scores and neuropsychological performance, while GAF scores were positively correlated with executive functions and processing speed.

**Table 2:** Demographic and clinical variables of AR-PP-well, AR-PP-unwell and NR

	AR-PP-well *n* = 25	AR-PP-unwell *n* = 18	NR *n* = 48	Statistics	*p*-value
Age (years), m (SD)	33.3 (5.5)	30.9 (5.9)	32.9 (4.6)	F = 0.994	0.380
Ethnicity (Caucasian), n (%)	18 (72%)	9 (50%)	38 (79.2%)	χ²(2) = 5.46	0.065
Parity (primiparity), n (%)	17 (68%)	10 (55.5%)	25 (52.1%)	χ²(3) = 8.62	0.071
Education level Degree/diploma Vocational/college or lower	20 5	12 6	40 8	χ²(2) = 2.22	0.330
Diagnosis	23 BD, 1 PP, 1 SZA	11 BD, 4 SZA, 3 PP	N/A	N/A	N/A
Medications (yes), n (%)^a^	13 (52%)	11 (61.1%)	N/A	χ²(2) = 0.667	0.414
Antipsychotic daily dose mg (CPZ equivalents), m (SD) ^a^	222 (303)	375 (292)	N/A	U = 17.0	0.073
PANSS Total score, m (SD) ^b^	32.1 (8.7)* **	38.3 (3.5)*	30.4 (1.1)	K = 30.88	**< 0.001**
YMRS Total score, m (SD) ^b^	1.4 (2.6)	2.5 (2.2)*	0.65 (1.3)	K = 11.6	**0.003**
HAM-D Total score, m (SD) ^b^	4.8 (5.8)**	9.1 (4.5)*	2.8 (2.5)	K = 21.56	**< 0.001**
CGI Total score, m (SD) ^b^	2.4 (0.8)*	3.0 (0.7)*	2.0 (0.0)	K = 35.72	**< 0.001**
GAF Total score, m (SD) ^b^	68.8 (8.4)*	77.6 (10.4)*	91.5 (4.2)	K = 57.191	**< 0.001**

AR-PP: women at risk of post-partum psychosis; BD: bipolar disorder; BRIEF PSY; brief psychotic episode: CGI: clinical global impression; GAF: global assessment of functioning; HAM-D: Hamilton Depression Rating Scale; m: mean; N/A: not applicable; NR: women not at risk; PANSS: Positive and Negative Syndrome Scale; PP: previous episode of postpartum psychosis; SD: standard deviation; SZA: schizoaffective disorder; YMRS: Young Mania Rating Scale

^a^ third trimester

^b^ baseline (25–30 weeks gestation)

*p*-values refer to the comparison between AR-PP-well, AR-PP-unwell and NR

* Significant results of post-hoc analyses between AR-PP-well and NR and between AR-PP-unwell and NR

** Significant results of post-hoc analyses between AR-PP-well and AR-PP-unwell

Higher PANSS, YMRS, HAM-D and CGI scores indicate more severe symptoms, while higher GAF scores indicate better functioning

### Differences in neuropsychological performance between women at risk of PD, women at risk of PP and NR

After controlling for education level, we found no differences in premorbid IQ (WTAR) between the three groups. Full-scale IQ was lower in women at risk of PP compared to women at risk of PD (F = 3.31, *p* = 0.04), but there was no difference between women at risk of PD or women at risk of PP and NR. The three groups differed in verbal learning and memory (F = 3.75, *p* = 0.026), visual memory (χ² Wald = 16.8, *p* < 0.01), executive function (F = 7.12, *p* = 0.001), verbal comprehension (χ² Wald = 8.9, *p* = 0.012) and processing speed (χ² Wald = 9.2, *p* = 0.01) (Table 3). Bonferroni-Holm post-hoc analyses revealed that this was due to women at risk of PP presenting with worse executive function (*p* = 0.007) and processing speed (*p* = 0.011) compared to NR. Compared to women at risk of PD, women at risk of PP also presented worse scores in verbal learning and memory, visual memory, executive function, verbal comprehension and processing speed (all p’s < 0.025, Table S2). The performance of women at risk of PD did not differ from that of NR in any domain. Therefore, we did not test for differences in neuropsychological performance between women at risk of PD who experienced a relapse in the post-partum (*n* = 6) and women who remained well (*n* = 47).

**Table 3:** Differences in neuropsychological performance between AR-PD, AR-PP and NR

	AR-PD *n* = 53	AR-PP *n* = 43	NR *n* = 48	Statistics	*p*-value
FSIQ, mean (SD)	100 (16.9)**	96.7 (14.1)	104.0 (15.0)	F = 3.31	**0.040**
IQ (WTAR), m (SD)	106 (16.2)	104 (15.0)	108 (14.5)	χ² Wald = 5.46	0.065
Verbal learning and memory, m (SD)	-0.212 (0.93)**	-0.536 (1.17)	-0.026 (0.97)	F = 3.75	**0.026**
Visual memory, m (SD)	0.080 (0.81)**	-0.481 (1.02)	-0.0002 (0.90)	χ² Wald = 16.8	**< 0.001**
Executive functions, m (SD)	-0.262 (1.01)**	-0.752 (1.18)*	0.009 (0.90)	F = 7.12	**0.001**
Verbal comprehension, m (SD)	-0.018 (1.24)**	-0.278 (0.93)	-0.040 (1.13)	χ² Wald = 8.9	**0.012**
Processing speed, m (SD)	-0.194 (0.84)**	-0.555 (1.01)*	0.070 (0.76)	χ² Wald = 9.2	**0.010**

AR-PD: women at risk of perinatal depression; AR-PP: women at risk of post-partum psychosis; FSIQ: full scale intelligence quotient; IQ: intelligence quotient; m: mean; NR: women not at risk; SD: standard deviation; WTAR: Wechsler Test of Adult Reading

All values except FSIQ and IQ are expressed as z-scores. A negative z-score indicates that the score is below the normative standards for the control sample

*p*-values refer to the comparison between AR-PD, AR-PP and NR

* Significant results of post-hoc analyses between AR-PD and NR and between AR-PP and NR

** Significant results of post-hoc analyses between AR-PD and AR-PP

### Differences in neuropsychological performance in women at risk of PP who experienced a relapse in the post-partum

Full-scale IQ was lower in women at risk PP-unwell compared to those NR (*p* = 0.008). There was a significant difference between women at risk of PP-well, women at risk of PP-unwell and NR in verbal learning and memory (F = 3.791, *p* = 0.032), visual memory (K = 6.09, *p* = 0.048), executive functions (F = 6.411, *p* = 0.004) and processing speed (K = 11.75, *p* = 0.003) (Table 4). Tukey post-hoc analyses revealed that the women at risk of PP-unwell presented worse scores in verbal learning and memory (*p* = 0.003), visual memory at a trend level (*p* = 0.056), executive functions (*p* = 0.001) and processing speed (*p* = 0.003) compared to NR. Of note, women at risk of PP-well had better verbal learning and memory (*p* = 0.035) compared to women at risk of PP-unwell (Table S3).

**Table 4:** Differences in neuropsychological performance between AR-PP-well, AR-PP-unwell and NR

	AR-PP-well *n* = 25	AR-PP-unwell *n* = 18	NR *n* = 48	Statistics	*p*-value
FSIQ, mean (SD)	101 (12.0)	91.5 (15.4)*	104.0 (15.0)	F = 4.019	**0.025**
IQ (WTAR), m (SD)	107 (11.8)	99.4 (17.9)*	108 (14.5)	K = 4.13	0.127
Verbal learning and memory, m (SD)	-0.207 (0.92)**	-1.05 (1.35)*	-0.026 (0.97)	F = 3.791	**0.032**
Visual memory, m (SD)	-0.349 (0.96)	-0.665 (1.10)	0.0002 (0.90)	K = 6.09	**0.048**
Executive functions, m (SD)	-0.519 (1.13)	-1.06 (1.19)*	0.009 (0.90)	F = 6.411	**0.004**
Verbal comprehension, m (SD)	-0.032 (0.78)	-0.620 (1.03)	-0.040 (1.13)	K = 5.15	0.076
Processing speed, m (SD)	-0.255 (0.85)	-0.978 (1.09)*	0.070 (0.76)	K = 11.75	**0.003**

AR-PP: women at risk of post-partum psychosis; FSIQ: full scale intelligence quotient; IQ: intelligence quotient; m: mean; NR: women not at risk; SD: standard deviation; WTAR: Wechsler Test of Adult Reading

All values except FSIQ and IQ are expressed as z-scores. A negative z-score indicates that the score is below the normative standards for the control sample

*p*-values refer to the comparison between AR-PP-well, AR-PP-unwell and NR

* Significant results of post-hoc analyses between AR-PP-unwell and NR

** Significant results of post-hoc analyses between AR-PP-well and AR-PP-unwell

## Discussion

To the best of our knowledge, this is the first study that compared neuropsychological performance in the third trimester of pregnancy in women at risk of PD and PP, and in women not at risk of psychiatric disorders. Our main finding is that women at risk of PP presented worse executive function and processing speed compared to NR and lower performance in all cognitive domains compared to women at risk of PD. In turn, women at risk of PD did not show any difference with NR. We also found evidence of impaired neuropsychological performance in pregnancy in women at risk of PP who developed a psychiatric relapse in the first four weeks post-partum relative to NR. Interestingly, women at risk of PP who remained well in the first four weeks post-partum presented neuropsychological performance that lay between that of women NR and those who developed symptoms.

In line with our hypothesis, we found that women at risk of PP had worse neuropsychological performance compared to NR, in particular in the executive function and processing speed domains. Deficits in executive function, the set of abilities that control behavioral responses (Diamond 2013), have been consistently reported in individuals with psychosis (Orellana and Slachevsky 2013; Thai et al. 2019) and BD (Huang et al. 2023; Xu et al. 2012). Similarly, meta-analytic evidence has highlighted processing speed deficits as central to cognitive impairment in psychosis (Dickinson et al. 2007; Knowles et al. 2010) and BD (Bora et al. 2010). Furthermore, our finding that women at risk of PP had worse performance compared to women at risk of PD in all cognitive domains is consistent with a large body of research showing that individuals with psychosis present poorer cognitive ability compared to individuals with MDD (Bogie et al. 2024; Trapp et al. 2017). Interestingly, 31 women at risk of PD (58.5%) presented with a current MDD, while women at risk of PP did not show depressive symptoms (HAM-D score = 6.5 ± 5.1) (Zimmerman et al. 2013). This finding suggests that neuropsychological performance in pregnancy is not influenced by the presence of acute depressive symptoms.

Notably, one study conducted in pregnant women with no history of mental health problems found that high levels of dehydroepiandrosterone correlated with better visuospatial performance, verbal episodic memory, attention and fewer psychotic symptoms, while higher progesterone levels were associated with psychotic symptoms and confusion (Galen Buckwalter et al. 1999). This might be explained by the fact that sex hormones, in particular estradiol and progesterone, modulate dopamine synthesis and release, and modify the basal firing rate of dopaminergic neurons (Taylor et al. 2023). Crucially, preclinical and clinical investigations suggest that dopamine plays a key role in cognitive processes, and disruptions in the dopaminergic system are thought to be involved not only in the etiology of psychotic disorders, but also in cognitive impairment (Conn et al. 2020). Thus, the dramatic changes in estradiol and progesterone levels that occur during pregnancy may contribute to dopamine alterations, which in turn might affect neuropsychological performance. Although we did not test sex hormones, it is intriguing to speculate whether the interplay between sex hormones and dopamine affects cognitive performance more significantly in women at risk of PP than in women at risk of PD or those not at risk of mental health problems. Future studies testing the relationship between sex hormones, dopamine and cognition in women at risk for perinatal psychopathology are needed to clarify this aspect. In addition to sex hormones and dopamine, oxytocin and cortisol might also be implicated in cognitive dysfunctions in women at risk of PP, but existing evidence does not allow to draw any firm conclusions (Palacios-Hernández et al. 2024).

Differently from women at risk of PP, women at risk of PD did not show any difference in neuropsychological performance from NR. This is somewhat unexpected, as there is ample evidence of impairment in processing speed, learning and memory in individuals with depression (Kriesche et al. 2023). However, these deficits seem less pronounced in remission phases and tend to become more apparent with age and with an increasing number of depressive episodes (Kriesche et al. 2023), which might explain why we did not observe them in our sample of young women that included also a large proportion of participants with MDD in remission (*n* = 22, 41.5%). Furthermore, our negative findings might be explained by the fact that women with a current MDD in our study presented only mild depressive symptoms (HAM-D score = 11.4 ± 7.14), while deficits in cognitive performance seems to be associated with severe depressive symptoms (McDermott and Ebmeier 2009). Importantly, the few available studies on cognition in women with PD highlight the presence of a heterogeneous picture, with investigations reporting an association between maternal mood and cognitive changes during pregnancy (Hampson et al. 2015; Kataja et al. 2017; Xu et al. 2023) and others showing no correlation (Liakea et al. 2022), indicating that more research is needed to clarify whether depression in the perinatal period is associated to changes in neuropsychological performance.

Importantly, we found that women at risk of PP who went on to develop a psychiatric relapse in the first four weeks post-partum showed lower full-scale IQ and poorer verbal learning and memory, visual memory, executive functions and processing speed in pregnancy compared to NR, independently of symptoms severity. Cognitive dysfunction is considered one of the greatest predictors of functional outcome in individuals with psychosis (Green et al. 2004). Previous evidence has suggested that lower IQ can predict psychotic relapse and withdrawal from research studies in people with psychosis (Di Michele et al. 2007; Fond et al. 2019). In addition, recent reports suggest that both generalized and specific cognitive deficits represent early markers of relapse in psychosis (Cuesta et al. 2022; Tao et al. 2023). Our findings are in line with evidence on cognitive deficits in psychosis unrelated to pregnancy and indicate that neuropsychological impairments in women at risk of PP who develop clinically meaningful symptoms in the post-partum might represent a biomarker that could potentially help identify women at risk most likely to relapse. If replicated, these findings hold the potential to guide preventive therapeutic interventions.

Lastly, we observed that women at risk of PP who remained well in the first four weeks post-partum presented neuropsychological performance that lay between those of women at risk of PP who became unwell and NR. Interestingly, this result aligns with evidence from functional magnetic resonance imaging studies on two different samples of women at risk of PP. In particular, one task-based study showed that women at risk of PP who remained well had an intermediate pattern of brain activity and functional connectivity during a working memory task compared to women at risk who developed a PP episode and controls (Kowalczyk et al. 2021). Similarly, women at risk of PP who remained well displayed intermediate functional connectivity within the triple network (i.e., executive, salience and default mode networks), a system involved in controlling higher cognitive and affective functions (Sambataro et al. 2021). Overall, these findings suggest that functional brain activity patterns may act as compensatory neural mechanisms that protect women at risk from developing PP and could potentially be associated with better cognitive performance. However, in our study, women at risk of PP who remained well in the first four weeks post-partum presented better performance only in one domain comparted to women at risk of PP who relapsed. There results must therefore be interpreted with caution and future studies should further investigate the presence of differences in cognitive profiles between women at risk of PP who experience a relapse in the post-partum and women who do not.

Our study has a number of strengths. First, women at risk of postpartum psychopathology represent a difficult clinical population to recruit and follow up, especially closer to time of delivery, as they often struggle with existing or lifetime mental health problems, in addition to potential anxiety related to the arrival of the baby or to the consequences of ongoing pharmacological treatment. Here, we were able to study this difficult to recruit population and to conduct a detailed clinical and neuropsychological assessment. Furthermore, the inclusion of a comparison group of women with no perinatal mental health problems and in the same perinatal period limits the possibility that any difference identified could be related to hormonal changes.

Notwithstanding these strengths, it should be also considered that our sample size is relatively small. As a result, we were not able to test for neuropsychological differences between women at risk of perinatal psychopathology that presented symptoms in pregnancy and those who did not because of the limited sample size of the subgroups. Future studies with larger samples could evaluate the specific relationship between neuropsychological function and symptom severity in pregnancy. Similarly, the neuropsychological assessment was only performed during pregnancy and not in the post-partum, and therefore we were not able to investigate the persistence of the differences we identified in pregnancy. Furthermore, the group of women at risk of PD is diagnostically heterogenous, as it includes women with a current episode of depression, as well as women with a history of previous depression. Since these states may differ in clinical course, it is also possible that neuropsychological performance in these subgroups differ significantly. An additional potential limitation is that women were not drug naïve or drug free, with 33 taking medications during the third trimester of pregnancy. This is important, as there is ample evidence suggesting that antipsychotics, mood stabilizers and antidepressants could potentially affect neuropsychological performance (Haddad et al. 2023; Liu et al. 2021; Wingo et al. 2009). However, we believe this is unlikely, as we found no correlation between antipsychotic dose and neuropsychological performance and no differences in antipsychotic daily dose in the third trimester of pregnancy between women at risk of PP who became unwell and those who remained well. In addition, only a limited number of AR-PP women were taking mood stabilizers (*n* = 5) and antidepressants (*n* = 5), thus reducing the potential confounding effect of these drugs on cognitive performance. Finally, we were unable to test the effect of hormonal level changes (i.e., estrogen, progesterone, cortisol, oxytocin and thyroid hormones) on neuropsychological performance. Future studies on the association between hormonal changes in pregnancy and in the post-partum and cognitive ability are warranted to clarify this relationship.

In conclusion, this study represents the first step towards achieving a better understanding of neuropsychological changes in pregnancy in women at risk of postpartum psychopathology. Our results that women at risk of PP presented worse neuropsychological performance compared to women at risk of PD and NR might be explained by the complex interplay between sex hormones, dopamine, oxytocin and cortisol. In addition, our findings that women at risk of PP who developed a psychiatric relapse in the first four weeks post-partum showed worse neuropsychological performance in pregnancy compared to NR and women who remained well suggests that cognition could be further studied as phenotype for the disease, with a view to help guiding earlier therapeutic interventions in the future. To clarify this aspect, future studies with larger sample sizes and a longitudinal design and are warranted.

## Electronic supplementary material

Below is the link to the electronic supplementary material.

Supplementary Material 1

## Data Availability

Due to privacy and ethical concerns, the data generated during this study can be provided by the corresponding author upon reasonable request and with necessary approvals.
