# Supplementary material for: Neuropsychological performance in women at risk of postpartum depression and postpartum psychosis
Source: Arch Womens Ment Health. 2024 Aug 31;28(1):55–65. doi: 10.1007/s00737-024-01510-9 (PMC11762223; doi:10.1007/s00737-024-01510-9)
Supplement: Supplementary file 1 — Supplementary Material 1 [file 737_2024_1510_MOESM1_ESM.docx]

**Neuropsychological performance in women at risk of postnatal depression and postpartum psychosis**

*Supplementary Material*

**Supplementary Methods**

**Exclusion criteria**

Exclusion criteria were: a) uterine anomaly; b) known obstetric complications in the index pregnancy; c) severe or relevant chronic medical conditions, such as cardiovascular disease, metabolic or endocrine disorder. Women at risk of postpartum depression (PD) only because of a history of major depressive disorder (MDD) were additionally excluded if they: a) presented any current DSM-IV diagnosis other than co-morbid anxiety disorder; b) had a past history of psychosis or bipolar disorder; c) were taking antidepressants at baseline. Women not at risk (NR) had to be negative for any personal history of mental health problems and a family history of postpartum psychosis (PP).

**Sample**

A total of 144 women were included in the current analyses (At risk of PP n=43, At risk of PD n=53, NR n=48). In the first four weeks post-partum, 18 women at risk of PP developed a psychiatric relapse (At risk of PP-unwell) and 25 remained well (At risk of PP-well). In the PP-unwell group, 11 women had a history of BD, four of SZA, 3 of previous PP; in the PP-well group 23 women had a history of BD, 1 of SZA and 1 of previous PP.

**Definition of postpartum psychosis**

Women at risk of PP were followed up to establish the presence of any psychiatric relapse if, in the first 4 weeks postpartum, they either a) met DSM-IV diagnostic criteria for major depressive disorder, bipolar disorder, schizoaffective disorder or Psychosis NOS; or b) had a combination of DSM-IV symptoms that did not meet diagnostic criteria but impacted their daily functioning (e.g., their ability to care for the baby or themselves) and were of sufficient intensity to require a change in treatment (either pharmacological or management plan) (Wesseloo et al. 2016). This broader definition was considered appropriate because all the women at risk of PP were closely monitored by perinatal psychiatric services and most of them took psychiatric medications to prevent the onset of PP or to treat the symptoms as soon as they developed to prevent them from worsening. This 4-week timeframe was chosen according to the DSM-IV postpartum-onset specifier (American Psychiatric Association 1994).

**Neuropsychological assessment**

At 30 weeks of pregnancy (30.2 ± 3.7, range: 21.7 - 39.1 weeks), we evaluated seven neurocognitive domains, using the Wechsler Adult Intelligence Scale – Revised (WAIS-R) (Wechsler 1981), Wechsler Test of Adult Reading (WTAR) (Wechsler 2001) and Wechsler Memory Scale-III (WMS-III) (Wechsler 1997). These included: full-scale intelligence quotient (FSIQ), premorbid IQ (WTAR), verbal learning and memory (Logical Memory Immediate and Delayed recall from the WMS-III), visual memory (Visual Reproduction Immediate and Delayed recall of the WMS-III), executive functions (Trail Making Test B, Verbal Fluency from the WMS-III), verbal comprehension (Similarities and Vocabulary from WAIS-III) and processing speed (Trail Making Test A and Digit symbol from the WAIS-III). For a detailed description of the scales see Strauss et al. (2006) (Strauss et al. 2006).

**Medications in pregnancy in women at risk of PP**

A total of 21 AR-PP women (48.8%) were taking psychotropic medications in the third trimester of pregnancy. Among these, 17 AR-PP women (40%, 8 AR-PP-unwell, 9 AR-PP-well) were taking antipsychotics, 5 AR-PP women (12%, 2 AR-PP-unwell, 3 AR-PP well) were taking mood stabilizers and 5 AR-PP women (12 %, 4 AR-PP-unwell, 1 AR-PP-well) were taking antidepressants. Women were taking the following medications: quetiapine (n=9), olanzapine (n=6), aripiprazole (n=1) and risperidone (n=1); lithium (n=2) and lamotrigine (n=3); sertraline (n=2), mirtazapine (n=2) and escitalopram (n=1)

**Supplementary Results**

**Postpartum clinical status in women at risk of PP according to the medication status in pregnancy**

Among the 21 AR-PP women who were taking medications during the third trimester of pregnancy, 12 remained well and 9 had a relapse in the post-partum. Differently, of the AR-PP women who were not taking medications in third trimester of pregnancy, 13 remained well and 9 presented symptoms in the post-partum.

**Correlations between clinical scales and neuropsychological performance**

In the overall sample, PANSS, YRMS, HAM-D and CGI scores did not correlate with neuropsychological performance, while GAF scores presented a positive correlation with verbal learning and memory, executive functions and processing speed (all p’s<0.001) (Tab. S.1). PANSS scores presented a positive correlation with YRMS, HAM-D and CGI scores (all p’s<0.001) and a negative correlation with GAF scores (p<0.001), YMRS scores presented a positive correlation with PANSS, HAM-D and CGI scores (all p’s<0.001) and a negative correlation with GAF scores (p<0.001), CGI scores presented a positive correlation with PANSS, YMRS and HAM-D (all p’s<0.001) and a negative correlation with GAF scores (p<0.001). All neuropsychological performance scores were positively correlated with each other (all p’s<0.001).

**Table S1. Correlations between clinical scales and neuropsychological performance in the overall sample**

|  |  | **PANSS Total score** | **YRMS Total score** | **HAM-D Total score** | **CGI Total score** | **GAF Total score** | |
| --- | --- | --- | --- | --- | --- | --- | --- |
| Verbal learning and memory | rho | -0.198 | -0.237 | 0.037 | -0.129 | | 0.272 |
|  | p-value | 0.052 | 0.056 | 0.671 | 0.138 | | **< 0.001** |
| Visual memory | rho | 0.040 | -0.003 | 0.045 | 0.004 | | 0.149 |
|  | p-value | 0.644 | 0.976 | 0.600 | 0.960 | | 0.079 |
| Executive functions | rho | -0.108 | -0.107 | -0.072 | -0.083 | | 0.343 |
|  | p-value | 0.232 | 0.232 | 0.424 | 0.358 | | **< 0.001** |
| Verbal comprehension | rho | -0.168 | -0.196 | -0.152 | -0.080 | | 0.203 |
|  | p-value | 0.047 | 0.060 | 0.071 | 0.346 | | 0.015 |
| Processing speed | rho | -0.089 | -0.060 | -0.122 | -0.043 | | 0.297 |
|  | p-value | 0.312 | 0.492 | 0.165 | 0.630 | | **< 0.001** |

CGI: clinical global impression; GAF: global assessment of functioning; HAM-D: Hamilton Depression Rating Scale; PANSS: Positive and Negative Syndrome Scale; YMRS: Young Mania Rating Scale.

**Table S2. Post-hoc analyses of differences in neuropsychological performance between AR-PD, AR-PP and NR**

|  |  | **AR-PD** | **AR-PP** | **NR** |
| --- | --- | --- | --- | --- |
| **FSIQ** | | | | |
| **AR-PD** | Mean difference | — | 6.63 | 1.96 |
|  | p-value | — | **0.039** | 1.00 |
| **AR-PP** | Mean difference |  | — | -4.67 |
|  | p-value |  | — | 0.249 |
| **IQ (WTAR)** | | | | |
| **AR-PD** | Mean difference | — | 5.90 | 3.09 |
|  | p-value | — | 0.059 | 0.442 |
| **AR-PP** | Mean difference |  | — | -2.80 |
|  | p-value |  | — | 0.442 |
| **Verbal learning and memory** |  |  |  |  |
| **AR-PD** | Mean difference | — | 0.502 | 0.144 |
|  | p-value | — | **0.025** | 0.444 |
| **AR-PP** | Mean difference |  | — | -0.357 |
|  | p-value |  | — | 0.135 |
| **Visual memory** | | | | |
| **AR-PD** | Mean difference | — | 0.7129 | 0.3059 |
|  | p-value | — | **< 0.001** | 0.056 |
| **AR-PP** | Mean difference |  | — | -0.4069 |
|  | p-value |  | — | 0.056 |
| **Executive functions** | | | | |
| **AR-PD** | Mean difference | — | 0.6917 | 0.0855 |
|  | p-value | — | **0.002** | 0.682 |
| **AR-PP** | Mean difference |  | — | -0.6062 |
|  | p-value |  | — | **0.007** |
| **Verbal comprehension** | | | | |
| **AR-PD** | Mean difference | — | 0.4991 | 0.3952 |
|  | p-value | — | **0.012** | 0.069 |
| **AR-PP** | Mean difference |  | — | -0.1039 |
|  | p-value |  | — | 0.569 |
| **Processing speed** | | | | |
| **AR-PD** | Mean difference | — | -0.46 | -0.10 |
|  | p-value | — | **0.025** | 0.553 |
| **AR-PP** | Mean difference |  | — | -0.56 |
|  | p-value |  | — | **0.011** |

AR-PD: women at risk of postnatal depression; AR-PP: women at risk of postpartum psychosis; FSIQ: full-scale intelligence quotient; IQ: intelligence quotient; NR: women not at risk; WTAR: Wechsler Test of Adult Reading. Results are corrected using a Bonferroni-Holm correction.

**Table S3. Post-hoc analyses of differences in neuropsychological performance between AR-PP-well, AR-PP-unwell and NR**

|  |  | **AR-PP-well** | **AR-PP-unwell** | **NR** |
| --- | --- | --- | --- | --- |
| **FSIQ** | | | | |
| **AR-PP-well** | Mean difference | — | 9.02 | -3.10 |
|  | p-value | — | 0.110 | 0.659 |
| **AR-PP-unwell** | Mean difference |  | — | -12.12 |
|  | p-value |  | — | **0.008** |
| **IQ (WTAR)** | | | | |
| **AR-PP-well** | Mean difference | — | 1.81 | -1.32 |
|  | p-value | — | 0.406 | 0.619 |
| **AR-PP-unwell** | Mean difference |  | — | -2.72 |
|  | p-value |  | — | 0.133 |
| **Verbal learning and memory** | | | | |
| **AR-PP-well** | Mean difference | — | 0.841 | -0.181 |
|  | p-value | — | **0.035** | 0.765 |
| **AR-PP-unwell** | Mean difference |  | — | -1.022 |
|  | p-value |  | — | **0.003** |
| **Visual memory** | | | | |
| **AR-PP-well** | Mean difference | — | 1.25 | -2.16 |
|  | p-value | — | 0.649 | 0.279 |
| **AR-PP-unwell** | Mean difference |  | — | -3.26 |
|  | p-value |  | — | 0.056 |
| **Executive functions** | | | | |
| **AR-PP-well** | Mean difference | — | 0.543 | -0.528 |
|  | p-value | — | 0.219 | 0.123 |
| **AR-PP-unwell** | Mean difference |  | — | -1.071 |
|  | p-value |  | — | **0.001** |
| **Verbal comprehension** | | | | |
| **AR-PP-well** | Mean difference | — | 2.647 | -0.526 |
|  | p-value | — | 0.147 | 0.927 |
| **AR-PP-unwell** | Mean difference |  | — | -3.046 |
|  | p-value |  | — | 0.079 |
| **Processing speed** | | | | |
| **AR-PP-well** | Mean difference | — | 3.16 | -1.97 |
|  | p-value | — | 0.065 | 0.345 |
| **AR-PP-unwell** | Mean difference |  | — | -4.65 |
|  | p-value |  | — | **0.003** |

AR-PD: women at risk of perinatal depression; AR-PP: women at risk of postpartum psychosis; FSIQ: full-scale intelligence quotient; IQ: intelligence quotient; NR: women not at risk; WTAR: Wechsler Test of Adult Reading. Results are corrected using a Tukey correction.

**Table S4. Differences in neuropsychological performance between AR-PP medicated, AR-PP non medicated and NR**

|  | **AR-PP medicated**  **n=21** | **AR-PP non medicated**  **n=22** | **NR**  **n=48** | **Statistics** | **p-value** |
| --- | --- | --- | --- | --- | --- |
| FSIQ, mean (SD) | 97.1 (16.4) | 96.4 (11.6) | 104.0 (15.0) | F=2.65 | 0.082 |
| IQ (WTAR), m (SD) | 107 (14.0) | 101 (15.6) | 108 (14.5) | χ² Wald=4.56 | 0.103 |
| Verbal learning and memory, m (SD) | -0.713 (1.38) | -0.382 (0.95) | -0.026 (0.97) | F=2.37 | 0.108 |
| Visual memory, m (SD) | -0.561 (1.16) | -0.405 (0.90) | -0.0002 (0.90) | F=2.71 | 0.078 |
| Executive functions, m (SD) | -0.829 (1.36)* | -0.682 (1.01)* | 0.009 (0.90) | F=5.42 | **0.008** |
| Verbal comprehension, m (SD) | -0.186 (1.10) | -0.366 (0.74) | -0.040 (1.13) | χ² Wald=2.79 | 0.248 |
| Processing speed, m (SD) | -0.613 (1.31)* | -0.504 (0.69)* | 0.070 (0.76) | F=5.68 | **0.007** |

AR-PP: women at risk of post-partum psychosis; FSIQ: full scale intelligence quotient; IQ: intelligence quotient; m: mean; NR: women not at risk; SD: standard deviation; WTAR: Wechsler Test of Adult Reading.

All values except FSIQ and IQ are expressed as z-scores. A negative z-score indicates that the score is below the normative standards of the control sample.

p-values refer to the comparison between AR-PP medicated, AR-PP non medicated and NR.

* Significant results of post-hoc analyses between AR-PP medicated and NR and between AR-PP non medicated and NR

**References**

1. Wesseloo R, Kamperman AM, Munk-Olsen T, Pop VJM, Kushner SA, Bergink V. Risk of Postpartum Relapse in Bipolar Disorder and Postpartum Psychosis: A Systematic Review and Meta-Analysis. *Am J Psychiatry*. 2016;173(2):117-127. doi:10.1176/APPI.AJP.2015.15010124

2. American Psychiatric Association. *Diagnostic and Statistical Manual of Mental Disorders, 4th Ed.*; 1994. https://psycnet.apa.org/record/1994-97698-000. Accessed January 18, 2024.

3. Wechsler D. *The Wechsler Adult Intelligence Scale-Revised*. Psychological Corp. New York; 1981.

4. Wechsler D. *Wechsler Test of Adult Reading*. (The Psychological Corporation, ed.). San Antonio; 2001.

5. Wechsler D. *Wechsler Memory Scale*. (The Psychological Corporation., ed.). San Antonio; 1997.

6. Strauss E, Sherman E, Spreen O. *A Compendium of Neuropsychological Tests: Administration, Norms, and Commentary, 3rd Ed.* Oxford University Press; 2006.
